# The Value of Graft Implantation Sequence in Simultaneous Pancreas-Kidney Transplantation on the Outcome and Graft Survival

**DOI:** 10.3390/jcm10081632

**Published:** 2021-04-12

**Authors:** Hans-Michael Hau, Nora Jahn, Sebastian Rademacher, Elisabeth Sucher, Jonas Babel, Matthias Mehdorn, Andri Lederer, Daniel Seehofer, Uwe Scheuermann, Robert Sucher

**Affiliations:** 1 Department of Visceral, Transplantation, Vascular and Thoracic Surgery, University Hospital of Leipzig, 04103 Leipzig, Germany; sebastian.rademacher@medizin.uni-leipzig.de (S.R.); jonas.babel@medizin.uni-leipzig.de (J.B.); matthias.mehdorn@medizin.uni-leipzig.de (M.M.); andri.lederer@medizin.uni-leipzig.de (A.L.); daniel.seehofer@medizin.uni-leipzig.de (D.S.); uwe.scheuermann@medizin.uni-leipzig.de (U.S.); robert.sucher@medizin.uni-leipzig.de (R.S.); 2 Department of Visceral, Thoracic and Vascular Surgery, University Hospital and Faculty of Medicine Carl Gustav Carus, Technische Universität Dresden, 01307 Dresden, Germany; 3 Department of Anesthesiology and Intensive Medicine, University Hospital of Leipzig, 04103 Leipzig, Germany; nora.jahn@medizin.uni-leipzig.de; 4 Department of Gastroenterology, Section of Hepatology, University Hospital of Leipzig, 04103 Leipzig, Germany; elisabeth.sucher@medizin.uni-leipzig.de

**Keywords:** simultaneous pancreas-kidney transplantation, immunosuppression, graft order, sequence, outcome, survival

## Abstract

Background/Objectives: The sequence of graft implantation in simultaneous pancreas-kidney transplantation (SPKT) warrants additional study and more targeted focus, since little is known about the short- and long-term effects on the outcome and graft survival after transplantation. Material and methods: 103 patients receiving SPKT in our department between 1999 and 2015 were included in the study. Patients were divided according to the sequence of graft implantation into pancreas-first (PF, *n* = 61) and kidney-first (KF, *n* = 42) groups. Clinicopathological characteristics, outcome and survival were reviewed retrospectively. Results: Donor and recipient characteristics were similar. Rates of post-operative complications and graft dysfunction were significantly higher in the PF group compared with the KF group (episodes of acute rejection within the first year after SPKT: 11 (18%) versus 2 (4.8%); graft pancreatitis: 18 (18%) versus 2 (4.8%), *p* = 0.04; vascular thrombosis of the pancreas: 9 (14.8%) versus 1 (2.4%), *p* = 0.03; and delayed graft function of the kidney: 12 (19.6%) versus 2 (4.8%), *p* = 0.019). The three-month pancreas graft survival was significantly higher in the KF group (PF: 77% versus KF: 92.1%; *p* = 0.037). No significant difference was observed in pancreas graft survival five years after transplantation (PF: 71.6% versus KF: 84.8%; *p* = 0.104). Kidney graft survival was similar between the two groups. Multivariate analysis revealed order of graft implantation as an independent prognostic factor for graft survival three months after SPKT (HR 2.6, 1.3–17.1, *p* = 0.026) and five years (HR 3.7, 2.1–23.4, *p* = 0.040). Conclusion: Our data indicates that implantation of the pancreas prior to the kidney during SPKT has an influence especially on the early-post-operative outcome and survival rate of pancreas grafts.

## 1. Introduction

Simultaneous pancreas-kidney transplantation (SPKT) is an established therapy for patients with insulin-dependent diabetes mellitus, complicated by end-stage renal disease. Successful SPKT leads to euglycemia, which could slow the progression of diabetic microvascular and macrovascular complications and it improves survival rates and recipients’ quality of life compared with patients on dialysis or patients after kidney transplantation alone [1,2,3,4,5,6,7,8,9].

However, its success depends on several factors such as the profiles of donors and recipients, methods of implantation techniques, and graft harvesting such as effects of ischemia-reperfusion injuries (IRI) on graft damages [6,9,10,11].

In this context, pancreas transplantation is associated with a high incidence of post-operative complications and up to 15% graft losses within the first year after SPKT [6,9,10,11].

Implantation of the pancreas before the kidney seems reasonable to avoid prolonged cold ischemic time and subsequent ischemic reperfusion injury of the pancreas graft, especially since kidney grafts can tolerate cold ischemia better than pancreas grafts [12]. However, there is currently no consensus on the best sequence of graft implantation during SPKT and in most cases the choice of the order is only made by the surgeon.

Therefore, the aim of this study was to determine the impact of graft implantation order on the outcome and survival after SPKT. We analyzed post-transplant outcome characteristics, survival rates, and risk factors for graft failures in SPKT depending on graft implantation order.

## 2. Material and Methods

### 2.1. Data Collection and Study Population

After approval by the local ethics committee [AZ-Nr: 111-16-14032016] medical data from all patients undergoing SPKT at the University Hospital of Leipzig between 1999 and 2015 were retrospectively analyzed from a prospectively collected electronic data base.

Patients were divided into two groups according to the order of graft implantation: (1) pancreas first (PF) and (2) kidney first (KF). The transplantation order was determined based on the ischemia times and implantation time points from the transplantation protocols.

### 2.2. Outcome Measures

Special emphasis was placed on patient and graft characteristics, postoperative complications, metabolic outcomes, renal function, and causes of graft failure depending on graft implantation order.

Characteristics included donor and recipient age, gender, and body mass index (BMI, weight in kg/height in m^2^), cytomegalovirus (CMV)-status, donor cause of death, duration of insulin dependent diabetes mellitus, duration of dialysis, and time on the waiting list. Peri- and post-transplant data included information on cold ischemia time (CIT) and warm ischemia time (WIT) of the grafts, immunosuppressive therapy as well as organ graft function: Duration of operation, rates of re-operation, infectious complications, number of rejection episodes, and delayed graft function (DGF). CIT is defined as time the organ spent in cold preservation solution after removal from the donor. WIT is the time from cross-clamping until cold perfusion, plus the time of implantation (organ out of ice until reperfusion). Surgical complications were defined as the need for relaparotomy within the first three months after transplantation.

Acute rejection episodes were suspected if there was an abrupt increase in serum amylase/lipase and/or serum glucose levels, together with a significant drop in serum C-peptide level and/or increased serum creatinine levels and missing diuresis as well as abdominal pain associated with sonographic swelling of the graft. If possible, the diagnosis was confirmed from endoscopic biopsies of the duodenal segment of the graft. Biopsies of the kidney graft were performed to confirm rejection. Pancreatic biopsies were not performed. Treatment of acute cellular rejection consisted of pulsed steroids (500 mg methylprednisolone on three consecutive days) or administration of 8 mg per kg bodyweight anti-thymocyte globulin (ATG) in parallel with increased baseline immunosuppression.

DGF of the kidney was defined as the requirement of dialysis in the first week following transplantation [13].

Pancreas graft failure was defined as resumed insulin therapy, removed pancreas, re-transplantation, or patient death.

Kidney graft failure was defined as the need for dialysis, removed kidney, re-transplantation, or patient death.

Postoperative mortality was considered as in-hospital mortality in all cases.

Laboratory parameters of ischemia-reperfusion-injury: Peak of C-reactive protein (CRP, mg/L) and serum lipase (mmol/L) within the first three days; endocrine function: low-density lipoprotein (LDL)-cholesterol/high density lipoprotein (HDL)-cholesterol ratio, HbA1C (%), C-peptide (ng/mL), and renal function: Creatinine (mmol/L) and urea (mmol/L) were analyzed up to five years after transplantation.

### 2.3. Organ Procurement and Transplantation

The procurement and transplantation of pancreas and kidney allografts were performed according to international standards and guidelines as described previously [6,10,14,15,16,17,18].

In short, the pancreas was transplanted into the right iliac fossa using a standard technique with an intraperitoneal location in the right iliac fossa. The Y-graft was anastomosed to the recipient’s common iliac artery, the portal vein was connected to the inferior vena cava of the recipient. Exocrine drainage was carried out with a hand-sutured side-to-side duodenojejunostomy 40 cm beyond the flexure of Treitz [10,18]. The exocrine drainage was always accomplished immediately after reperfusion, to decrease the risk of donor duodenum distension and trigger of consecutive graft pancreatitis. The main reason why the kidney transplant was performed before the pancreas transplant was the possibility of working in two teams. One was responsible for the back-table preparation, one team was responsible for the recipient operation and transplant procedure. Since the back-table preparation of the kidney is less time consuming, the preparation of the kidney was always performed first. Once completed the kidney was immediately handed over to the implant surgeons for transplant.

### 2.4. Immunosuppression

Immunosuppressive therapy comprised an induction therapy with the interleukin-2 receptor antagonist basiliximab or antithymocyte globulin, followed by a triple maintenance immunosuppression consisting of calcineurin inhibitors (tacrolimus or cyclosporine), and/or antimetabolites (mycofenolate mofetil or sirolimus) and tapered steroids (prednisolone).

### 2.5. Statistical Analysis

Baseline data are presented as mean values with the standard deviation (SD) such as the proportion percentage (%). For comparison between the two groups, the appropriate statistical significance test including the Student’s *t*-test, χ2, analysis of variance (ANOVA), Kruskal–Wallis, and Wilcoxon–Mann–Whitney test was used. Survival rates were calculated using the Kaplan–Meier analysis and the log-rank test was applied to test statistical significance. Graft survival was calculated as the time from initial transplant to graft failure, censoring for death with a functioning graft, and grafts still functioning at time of analysis. Patient survival is defined as time from transplant to patient death, censoring for patients still alive at time of analysis. If a recipient was alive or lost to follow-up at time of last contact, then survival time was censored at time of last contact. Multivariate analysis was performed with logistic regression analysis. Variables to be entered into the multiple logistic regression analysis were chosen on the basis of the results of univariate analysis. *p* values < 0.05 were regarded as significant. All statistical analyses were performed by using IBM SPSS Statistics 24.0 (IBM Corporation, Armonk, NY, USA).

## 3. Results

### 3.1. Baseline Characteristics

The overall study population included 103 patients receiving SPKT in our department between 1999 and 2015. In 61 patients (59.2%), the pancreas was implanted before the kidney (PF), and in 42 patients (40.8%) the kidney was implanted first (KF). The mean follow-up period was 9.1 ± 1.2 years (PF: 9.1 ± 1.6 years versus KF: 9.2 ± 0.8 years, *p* = 0.949). Donor, recipient, and graft characteristics according to the different implantation order are summarized in Table 1. The two groups were similar in most of their transplant characteristics.

### 3.2. Outcome

The analysis of post-operative outcome parameters is shown in Table 2. In the overall study population, the most frequent complications were episodes of acute rejection and delayed kidney graft function. In a comparison of the two groups, delayed graft function of the kidney (*p* = 0.030), episodes of acute rejection (*p* = 0.034), rates of graft pancreatitis (*p* = 0.04), and total rate of vascular thrombosis of the pancreas (*p* = 0.03) were significantly higher in the PF group.

In total, 1.9% (*n* = 2) of the patients developed arterial thrombosis and 7.8% (*n* = 8) of the patients developed venous thrombosis. The majority of thrombosis (*n* = 8) occurred within four weeks after SPKT. All arterial thrombosis occurred in the PF group (*n* = 2). Thereby, in one patient the graft could be preserved with re-laparotomy and thrombectomy.

During the first year after SPKT acute rejection occurred in eleven patients (10.7%) in the PF group and in two patients (1.9%) in the KF group (*p* = 0.04). In total, acute rejection occurred in 26 patients (25%) during the complete follow-up period (PF: *n* = 20 versus KF: N = 6, *p* = 0.034). In 13 patients (50%) acute rejection could be confirmed histologically with renal biopsies. In one case (3.8%) the diagnosis was confirmed with endoscopic biopsies of the duodenal segment of the graft.

Within the first three days after transplantation, CRP peak—as an indicator of ischemia-reperfusion injury, such as peak of lipase—was also significantly higher in the PF group in comparison with the KF group (CRP, PF: 133.4 ± 7.9 mg/L versus KF: 104.1 ± 6.8 mg/L, *p* = 0.001; lipase, PF: 8.1 ± 4.1 mmol/L versus KF: 3.2 ± 3.5 mmol/L, *p* = 0.022).

Overall, in-hospital mortality was higher in the PF group (*n* = 5, 8.2%) compared with the KF group (*n* = 2, 4.8%) (*p* = 0.496). The causes of death included multiple organ failure (*n* = 2), septic shock (*n* = 2) and fatal heart attack (*n* = 1) in the PF group and septic shock (*n* = 1), and heart failure (*n* = 1) in the KF group, respectively.

### 3.3. Metabolic and Renal Function

With regard to renal function and LDL/HDL ratio, there were no significant differences between the two groups three months, one year, and five years after SPKT. Regarding the endocrine function of the pancreas, HbA1c levels tended lower for KF group by one and five years after SPKT but did not reach significance (*p* = 0.075 and *p* = 0.08, respectively) (Supplementary Table S1).

### 3.4. Short- and Long-Term Survival

**Figure 1:**
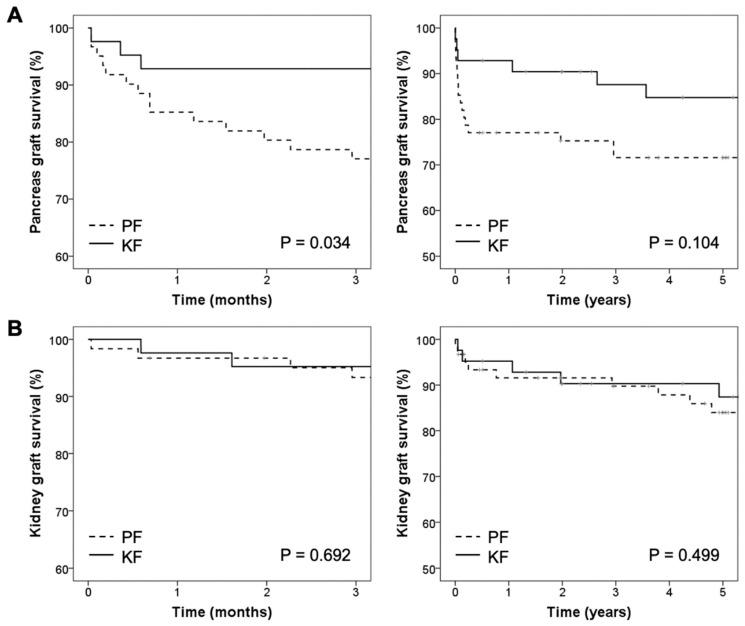
Graft and patient survival according to the graft implantation order. (**A**) pancreas graft survival, and (**B**) kidney graft survival three months and five years after simultaneous pancreas-kidney transplantation.

Pancreas graft survival was significantly higher when the kidney was implanted first. During the first three months after SPKT, the percentage of pancreas graft loss was 23% in the PF group and 7.9% in the KF group (*p* = 0.034). The one-, three-, and five-year pancreas graft survival rates in patients after SPKT were 75.3%, 71.6%, and 71.6% in the PF group, respectively, and 90.4%, 87.7%, and 84.8% in the KF groups, respectively (*p* = 0.104) (Figure 1A). The one-, three-, and five-year kidney graft survival rates in patients after SPKT were 90.0%, 90%, and 84% in the PF group, respectively, and 92.8%, 90.2%, and 87.3% in the KF group, respectively (*p* = 0.499) (Figure 1B). Overall patient survival after one, three, and five years was 88.5%, 86.8%, and 84.9%, respectively, in the PF group, and 92.9%, 92.9%, and 90.2%, respectively, in the KF group (*p* = 0.419).

Multivariate Cox regression analysis of the total study population revealed that donor cause of death, donor recipient age and recipient BMI, duration of pancreas cold ischemia time and order of graft implantation are independent predictors of pancreas graft loss within three months and five years after SPKT. Era of transplantation and recipient gender showed a significant impact on pancreas graft survival at three months only, while they had no significant effect on 5-year graft survival (Table 3).

## 4. Discussion

The current study showed that the implantation of the kidney graft before the pancreas graft during SPKT is associated with reduced rates of post-operative complications and significantly better pancreas graft survival in the early post-operative course.

The most frequent postoperative complication after SPKT and subsequent pancreas graft loss remain pancreas graft thrombosis. In our study, the average pancreas graft thrombosis rate was 9.7% (1.9% arterial and 7.8% venous, respectively), which is comparable to published data [19,20]. There are a number of well-described donor and recipient risk factors associated with thrombosis in pancreas transplantation, such as donor age and obesity, cause of death [19]. However, none of these risk factors were different between the two groups (Table 1). Furthermore, in our cohort rates of acute rejection, delayed graft kidney function as well as overall graft survival are comparable with previous publications [6,21].

To our knowledge, only two other studies have previously examined the effect of graft implantation order on short- and long-term outcomes in SPKT. In a retrospective single-center analysis of 151 patients after SPKT, Salzedas–Netto et al. showed a significantly higher three-month pancreas graft survival when the kidney was implanted first (pancreas graft survival in three months, PF: 74.1 versus KF: 89.4%, *p* = 0.022) [22]. In accordance with our data, post-operative complications primarily occurred in the PF group and had a particular influence on the early post-operative outcome. However, a further comparison to our study is virtually impossible due to lack of detailed data.

In contrast, in a 2016 published register (Scientific Registry of Transplant Recipients) data analysis of 12,700 patients by Niclauss et al., the rate of pancreas graft loss within three months after SPKT was significantly lower in the PF group (PF: 9.4% versus KF: 10.8%, *p* = 0.011). Additionally, the frequency of technical graft failures was significantly lower in this group (PF: 5.6% versus KF: 6.9%, *p* = 0.005) [23]. Beyond three months, no significant differences were observed in graft survival between the two groups.

The reasons for an increased rate of complications and early graft loss in the PF group of our study remains speculative. One reason could be the mechanical stress after graft positioning and surgical retractor adjustment for the consecutive kidney transplant procedure. Salzedas–Netto et al. assumed that implantation of the kidney before the pancreas graft could reduce the risk of intra-operative damage from retractors to the pancreas and pancreatic edema. Unfortunately, no details on intraoperative findings and rector problems are available to us, that could support this thesis. Furthermore, different surgical access routes and procedures were used in the studies. In the study by Salzedas–Netto et al., organs were implanted intra-peritoneally (pancreas), as well as extra-peritoneally (kidney) by two surgical teams. In our analysis and the study by Salzedas–Netto et al., all patients underwent systemic drainage with side-to-side enteric anastomoses [22]. In contrast, in the study by Niclauss et al., exocrine pancreas secretion drainage was realized either enterically or into the bladder. However, influence of bladder drainage (44.7% of PF and 37.1% of KF) on graft survival and association with complication rates (especially vascular thrombosis) were not examined [23]. We recently introduced an intraoperative no touch real time monitoring technique for kidney and pancreas graft parenchyma evaluation using hyperspectral imaging (HSI) [24,25]. We believe that this novel procedure might be a useful tool to investigate on our hypothesis that mechanical stress to the organ implanted first during transplantation of the second organ might affect graft performance. In this context, HSI is well suited to detect venous congestion which would predominantly be detected by decreased perfusion indices and increased organ hemoglobin indices of the affected organ.

Prolonged CIT has a negative impact on pancreas graft survival and frequency of post-operative complications [23,26,27,28,29]. Therefore, transplanting pancreas grafts first seems to be reasonable as pancreas grafts tolerate cold ischemia worse than kidneys [12,30]. The study by Niclauss et al. revealed total pancreas preservation times of 12.2 ± 0.1 in the pancreas first and 14.3 ± 0.1 in the kidney first group, which were significantly longer when compared to pancreas preservation times in our patient groups [23]. In our analysis, Cox regression analysis showed that prolonged pancreas CIT (above 12 h) is an independent risk factor for pancreas graft survival at three months and at five years after SPKT, while cold ischemia time of the kidney graft had no significant impact on pancreas graft survival (Table 3). In our analysis, total pancreas CIT was relatively short and did not show a significant difference between the two groups (CIT pancreas KF: 11.5 h versus PF: 10.5 h, *p* = 0.08). Moreover, the time difference between the implantation of both organs was lower in the KF group (PF: 2.3 h versus KF: 1.4 h, Table 1). The study by Niclauss et al. could demonstrate that when kidney grafts are implanted first, prolonged surgery time between kidney and pancreas graft implantation mainly above two hours is associated with reduced pancreas graft survival in comparison with patients in the PF group [23]. Both time factors (relatively short pancreas CIT and time gap between organ implantation) may have contributed to the good outcome in the KF group in our cohort.

### 4.1. Remote Ischemic Preconditioning—Potential Pathophysiology Linked to Implantation Sequence

Remote ischemic preconditioning (RIP) is the phenomenon whereby brief episodes of ischemia and reperfusion applied in distant tissues like the lower extremity render organs subject to transplantation, more resistant to ischemia. The underlying mechanism of RIP are not fully understood, however, experimental studies suggest that a combination of circulating mediators and neuronal signaling might be responsible for conditioning of the targeted organ [31,32]. Further clinical studies in kidney and lung transplant recipients suggest that RIP prior transplantation may be beneficial or at least not harmful to the transplanted organ [33].

In the event of simultaneous pancreas-kidney transplantation the successive implantation of both organs requires a sequential (two-step) temporary clamping of external iliac vessels and by nature, imitate the procedure of RIP for the second organ to be transplanted. The assumption that pancreatic allografts may benefit from RIP if transplanted second to a kidney is highly speculative but spurs further research.

### 4.2. Limiting Factors

There are some limiting factors of this study. First, the low number of patients in each group and the retrospective non-randomized design should be mentioned. Second, the long investigation period and different surgical teams restricted data evaluation, thus making further controlled and prospective studies necessary.

## 5. Conclusions

In conclusion, our results suggest that the sequence of graft implantation during SPKT influences the early post-operative course. In our analysis, KF patients seem to have a slight advantage compared with patients receiving PF during SPKT. We would recommend a kidney first transplant if two parallel working teams are available, one doing the back-table preparation, one doing the transplant procedure. If one team is responsible for both back-table and the transplant procedure we would recommend a pancreas transplant first, since the pancreas is more sensitive to ischemia and one or two hours might matter. However, we also recommend a careful surgical retractor adjustment not to compromise the pancreas allograft. Further investigation is required in larger series to determine how the order of graft implantation correlates with post-transplant graft function. Our two main floated hypotheses, that (1) mechanical stress may harm the first and (2) remote ischemic preconditioning may have beneficial effects on the second organ subject to transplantation. Both assumptions may stimulate further investigation.

## Figures and Tables

**Table 1:** Donor, recipient, and transplant characteristics.

Variables	PF (*n* = 61)	KF (*n* = 42)	*p*-Value
**Donor**			
Age, years	22 ± 1.7	23 ± 1.5	0.928
Gender, male/female	33/28	28/14	0.202
BMI, kg/m^2^	22.2 ± 0.5	23.1 ± 0.4	0.416
Cause of death (head trauma, SAH, stroke, anoxia, infection, other, unknown)	28, 14, 4, 8, 1, 2, 4	17, 11, 3, 5, 2, 2, 2	0.964
Recipient			
Age, years	43 ± 1.1	42 ± 1.5	0.787
Gender, male/female	33/28	24/18	0.76
BMI, kg/m^2^	24.7 ± 0.5	25.0 ± 0.7	0.842
Duration of Diabetes, years	27 ± 1.2	26.5 ± 1.2	0.589
Previous Dialysis	46	34	0.518
Duration of dialysis, months	34.9 ± 5.9	30.1 ± 3.8	0.521
Waiting time, months	8.7 ± 1.5	8.8 ± 1.1	0.970
Transplant			
Era, 1998–2006/2007–2015	34/29	28/14	0.309
CMV D+/R−	16	7	0.252
Cold ischemia time, hours			
Pancreas	10.5 ± 0.3	11.5 ± 0.4	0.08
Kidney	12.8 ± 0.4	10.1 ± 0.3	0.001
Warm ischemia time, minutes			
Pancreas	39.1 ± 1.6	36.5 ± 2.2	0.348
Kidney	33.2 ± 2.3	31.0 ± 1.9	0.785
Operating time, hours	5.1 ± 0.9	5.2 ± 0.8	0.789
Immunosuppression			
Induction therapy (ATG/ IL-2 RA/ none)	38/16/7	29/9/4	0.779
CNI (Tacrolimus/CsA)	56/5	39/3	0.843
AP drug (MMF/SRL/none)	51/9/1	36/5/1	0.890

Data are shown as mean ± SD. BMI, body mass index; AP drug, antimetabolite; ATG, anti-thymocyte globulin; CNI, calcineurin inhibitor; CMV, cytomegalovirus; CSA, cyclosporin; D+, donor positive; IL-2 RA, Interleukin-2 receptor antagonist; KF, kidney first; MMF, mycofenolate mofetil; PF, pancreas first; R+ recipient positive; SAH, subarachnoid hemorrhage; and SRL, sirolimus.

**Table 2:** Postoperative complications after simultaneous pancreas-kidney transplantation.

Variables	PF (*n* = 61)	KF (*n* = 42)	*p*-Value
Delayed graft function (%)			
Pancreas	3 (4.9)	2 (4.8)	0.978
Kidney	13 (21.3)	2 (4.8)	0.019
Acute rejection episodes, combinded (%)			
1st year	11 (18.0)	2 (4.8)	0.040
Total	20 (32.8)	6 (14.3)	0.034
Pancreatitis (%)	11 (18.0)	2 (4.8)	0.040
Vascular thrombosis (%)			
Pancreas, total	9 (14.8)	1 (2.4)	0.030
Artery	2 (3.3)	0 (0)	0.234
Vein	7 (14.5)	1 (2.4)	0.09
Anastomosic leakage (%)	1 (1.6)	0 (0)	0.400
Bleeding (%)	7 (11.5)	3 (7.1)	0.466
Re-operation (%)	25 (41.0)	13 (31.0)	0.300
CMV-infection (%)	19 (31.1)	14 (33.3)	0.815

**Table 3:** Multivariate Cox regression analysis of predictors of pancreas graft loss three months and five years after simultaneous pancreas-kidney transplantation.

Variables			Time after SPKT											
			3 Months						5 Years					
			Univariate Analysis			Multivariate Analysis			Univariate Analysis			Multivariate Analysis		
			HR	95% CI	*p*-Value	HR	95 CI	*p*-Value	HR	95 CI	*p*-Value	HR	95 CI	*p*-Value
Donor														
Age			1.07	1.03–1.11	0.001	1.05	1.02–1.09	0.002	1.057	1.02–1.09	0.002	1.052	1.01–1.09	0.004
Gender (male versus female)			2.4	0.75–7.1	0.1				2.3	0.87–6.32	0.09			
BMI			1.21	1.03–1.41	0.015	1.247	1.052–1.478	0.011	1.20	1.05–1.38	0.01	1.180	1.023–1.361	0.023
Cause of death (Non-trauma versus trauma)			12.67	1.65–97.6	0.01	10.755	1.376–84.082	0.018	6.9	1.57–30.96	0.01	11.8	1.532–92.01	0.024
Recipient														
Age			1.08	1.02–1.14	0.008	1.126	1.050–1.208	0.001	1.053	1.005–1.104	0.003	1.093	1.035–1.154	0.001
Gender (male versus female)			0.32	0.10–0.91	0.030	0.302	0.105–0.866	0.026	0.34	0.11–0.99	0.04	0.426	0.180–1.088	0.052
BMI			1.13	1.03–1.23	0.006	1.117	1.041–1.331	0.010	1.21	1.10–1.32	<0.001	1.195	1.078–1.325	0.001
Transplant														
Era (1998–2006 versus 2007–2015)			9.49	1.26–71.6	0.029	8.1	1.1–61.5	0.04	2.83	0.96–8.34	0.05			
Warm ischemia time														
	Pancreas		0.996	0.68–1.45	0.985				1.01	0.23–5.2	0.967			
	Kidney		1.1	0.75–1.79	0.495				1.08	0.21–6.42	0.89			
CIT, hours														
	Pancreas													
		0–8	Ref.		0.005	Ref		0.007	Ref.		0.001	Ref.		0.005
		8–12	0.58	0.04–9.36	0.71	0.56	0.03–8.93	0.681	0.88	0.14–5.29	0.889	1.03	0.17–62.3	0.601
		>12	8.6	1.13–64.99	0.03	7.97	1.05–60.43	0.045	5.82	1.34–25.17	0.018	5.43	1.24–23.69	0.024
	Kidney													
		0–8	Ref.		0.06				Ref.		0.029	Ref.		0.027
		8–12	0.56	0.05–6.29	0.65				0.21	0.03–1.23	0.08	0.19	0.03–1.17	0.074
		>12	2.97	0.39–22.59	0.29				1.47	0.43–5.07	0.53	1.4	0.41–4.95	0.568
Graft implantation order (PF versus KF)			3.7	1.06–12.96	0.038	2.6	1.3–17.1	0.026	2.3	0.92–5.96	0.09	3.7	2.1–23.4	0.04
Immunosuppression														
Induction therapy														
	None		Ref.		0.272				Ref.		0.342			
	ATG		0.42	0.11–1.59	0.205				0.58	0.16–2.08	0.408			
	IL–2 RA		0.89	0.22–3.56	0.870				1.1	0.29–4.18	0.880			
CNI (Tac versus CSA)			0.36	0.10–1.28	0.1				0.44	0.13–1.51	0.19			
AP drug														
	None		Ref.		0.319				Ref.		0.292			
	MMF		0.32	0.04–2.29	0.247				0.4	0.54–2.98	0.371			
	SRL		0.11	0.01–1.9	0.133				0.18	0.01–1.78	0.121			

95CI, 95% confidence interval; HR, hazard ratio; and Tac, tacrolimus.

## Data Availability

Our database contains highly sensible data which may provide insight in clinical and personnel information about our patients and lead to identification of these patients. Therefore, according to organizational restrictions and regulations these data cannot be made publically available. However, the datasets used and/or analyzed during the current study are available from the corresponding author on reasonable request.

## Supplementary Materials

The following are available online at https://www.mdpi.com/article/10.3390/jcm10081632/s1, Supplementary Table S1. Metabolic outcome during the first 5 years after simultaneous pancreas-kidney transplantation.

## Abbreviations

BMI	Body mass index
CIT	Cold ischemia time
CMV	Cytomegalovirus
DGF	Delayed graft function
KF	Kidney-first
MMF	Mycophenolate mofetil
PF	Pancreas-first
RIC	Remote ischemic conditioning
SD	Standard deviation
SPKT	Simultaneous pancreas-kidney transplantation
WIT	Warm ischemia time
HSI	Hyperspectral Imaging
RIP	Remote ischemic preconditioning
