# Supplementary material for: The Value of Graft Implantation Sequence in Simultaneous Pancreas-Kidney Transplantation on the Outcome and Graft Survival
Source: J Clin Med. 2021 Apr 12;10(8):1632. doi: 10.3390/jcm10081632 (PMC8070486; doi:10.3390/jcm10081632)
Supplement: Supplementary file 1 [file jcm-10-01632-s001.pdf]

Supplementary Table 1. Metabolic outcome during the first 5 years after simultaneous pancreas-kidney transplantation.

Variables	Time after SPKT								
	3 months			1 year			5 years		
	PF	KF	*p*-value	PF	KF	*p*-value	PF	KF	*p*-value
C-peptide, ng/ mL	2.3 ±1.9	2.9 ±1.7	0.289	1.9 ±0.3	1.5 ±0.5	0.342	1.0 ±0.17	0.9 ±0.15	0.811
HbA1c, %	5.8 ±0.15	5.6 ±0.2	0.178	6.0 ±0.2	5.5 ±0.2	0.075	6.4 ±0.8	5.6 ±0.1	0.080
Lipase, mmol/ L	1.28 ±0.8	0.69 ±0.7	0.07	1.03 ±1.6	0.58 ±0.19	0.205	0.82 ±0.5	0.65 ±0.13	0.114
Creatine, mmol/ L	125 ±7.3	126 ±8.2	0.980	130 ±12.4	123 ±6.4	0.643	123 ±11.2	150 ±1.6	0.220
Urea, mmol/ L	8.8 ±0.5	7.9 ±0.6	0.239	9.2 ±0.7	10.4 ±1.9	0.493	8.9 ±0.7	9.8 ±0.8	0.441
LDL/ HDL	2.1 ±1.1	1.9 ±0.8	0.207	1.9 ±0.8	1.7 ±0.6	0.8.25	2.1 ±1.3	1.9 ±0.9	0.748

Data are shown as mean ± SD. HbA1c, glycated hemoglobin; KF, kidney first; PF, pancreas first.
